# Correlation between serum vitamin levels and gestational diabetes mellitus

**DOI:** 10.3389/fendo.2025.1569654

**Published:** 2025-10-07

**Authors:** Ying Qin, Qinwen Song, Xiaoyuan Jiang, Yixin Su, Hua Chen, Xiao Ji, Shujing Xu

**Affiliations:** ^1^ Department of Obstetrics, Affiliated Hospital of Shandong University of Traditional Chinese Medicine, Jinan, China; ^2^ The First Clinical Medical College, Shandong University of Traditional Chinese Medicine, Jinan, Shandong, China; ^3^ Shandong Second Medical University, Binzhou, Shandong, China; ^4^ First People’s Hospital of Jinan, Jinan, Shandong, China

**Keywords:** gestational diabetes mellitus, vitamins, oxidative stress, immunomodulation, therapy

## Abstract

Gestational diabetes mellitus (GDM) is a common clinical complication during pregnancy, with its pathogenesis not yet fully elucidated. Vitamin D contributes to GDM pathogenesis by regulating pancreatic β-cell function, immune responses, and lipid metabolism. Vitamin D deficiency may contribute to GDM through these mechanisms. Vitamin E levels in GDM patients are lower than those in normal pregnant women, and its deficiency may increase the risk of GDM, potentially due to its antioxidant properties, although the specific mechanisms remain unclear. The relationship between vitamin A levels and GDM is controversial. Additionally, the occurrence of GDM is closely associated with one-carbon metabolism, involving folic acid (vitamin B9), vitamin B12, and vitamin B6. Deficiencies in these vitamins may lead to homocysteine metabolism disorders, thereby contributing to GDM. Vitamin B3 plays a protective role against GDM by regulating redox reactions. Vitamin C deficiency has also been linked to GDM. Furthermore, combined supplementation with vitamin C and iron has been shown to reduce the incidence of pregnancy-associated iron-deficiency anemia to some extent.

## 1 Introduction

Gestational diabetes mellitus (GDM) refers to glucose metabolism disorders occurring during pregnancy that do not meet the diagnostic criteria for overt diabetes mellitus (1). It is a common complication during pregnancy that can have short- and long-term adverse effects on maternal and offspring health, including gestational hypertension, postpartum hemorrhage, macrosomia, and neonatal hypoglycemia. Clinically, most pregnant women with GDM do not exhibit obvious symptoms such as polydipsia, polyphagia, polyuria, or weight loss.

Currently, China recommends oral glucose tolerance testing (OGTT) for pregnant women between 24 and 28 weeks of gestation. A fasting blood glucose level ≥5.1 mmol/L, or 1-hour and 2-hour postprandial blood glucose levels ≥10.0 mmol/L and ≥8.5 mmol/L, respectively, are diagnostic of GDM (2). Since hyperglycemia often exists before GDM diagnosis and late diagnosis at 24–28 weeks may delay intervention, early prevention, diagnosis, and treatment of GDM are essential.

GDM is thought to result from β-cell dysfunction caused by insulin resistance, imbalances in inflammatory and adipokine levels, placental oxidative stress, and mitochondrial dysfunction (3). Significant differences in the levels of multiple vitamins have been observed between GDM and non-GDM pregnant women (4–8). This suggests that vitamin levels and their metabolism may be associated with the pathogenesis of GDM. Investigating these relationships could offer novel strategies for early diagnosis, prevention, and treatment of GDM.

## 2 Methodology

### 2.1 Literature search and selection strategy

A comprehensive literature search was conducted to identify relevant studies for this review. We systematically searched several major electronic databases, including PubMed, Elsevier, SpringerLink, Wiley Online Library, and Google Scholar. The search was restricted to articles published between January 2015 and August 2025 to ensure the inclusion of current and relevant research. These databases were selected because they host a wide array of high-quality, peer-reviewed articles pertinent to the fields of medicine, nutrition and obstetrics. To identify relevant publications, we developed a search strategy using a combination of keywords, such as: (“Gestational Diabetes Mellitus” OR “GDM”) AND (“Vitamins” OR “vitamin A” OR “Retinol” OR “vitamin D” OR “vitamin E” OR “vitamin C” OR “B Vitamins” OR “Folic Acid” OR “Vitamin B12”) AND (“pathogenesis” OR “risk factor” OR “oxidative stress” OR “insulin resistance”).

### 2.2 Selection criteria

A predefined set of inclusion and exclusion criteria guided the selection of studies. We included original research articles, such as randomized controlled trials, cohort studies, case-control studies, and meta-analyses, that were published in English. The primary focus of the included literature had to be the association between one or more vitamin levels and Gestational Diabetes Mellitus, including investigations into the underlying mechanisms, prevention or management of the condition. Conversely, we excluded articles that were not peer-reviewed, such as preprints, editorials, commentaries, letters to the editor, and conference abstracts without a full paper. Existing review articles were also excluded to ensure our analysis was based on primary data, although their reference lists were reviewed for potentially relevant studies. Furthermore, studies not focused on GDM, case reports, and research with insufficient data were not considered for this review.

### 2.3 Fat-soluble vitamins and GDM

#### 2.3.1 Vitamin A

Vitamin A, also known as retinol, plays a role in maintaining epithelial integrity, promoting cell growth, inhibiting membrane lipid peroxidation, and regulating immune function. As a potent antioxidant, it helps protect cells from free radical damage. Reduced vitamin A levels may exacerbate oxidative stress, aggravating endothelial damage, chronic low-grade inflammation, insulin resistance, and pancreatic β-cell dysfunction, ultimately leading to GDM.

Ma (9) conducted a retrospective analysis of 230 pregnant women and found abnormal levels of vitamin A and oxidative stress markers in GDM patients. Logistic regression analysis identified vitamin A levels as an independent risk factor for adverse pregnancy outcomes in GDM patients (P < 0.05). Another study revealed that mid-gestation serum vitamin A levels were lower in GDM patients compared to normal pregnant women, and logistic regression indicated that serum vitamin A could serve as a protective factor against GDM (P < 0.05) (10). However, other studies, such as Xue (6), reported no statistically significant differences in serum vitamin A levels across early, mid, and late gestation between GDM and non-GDM groups (P > 0.05).

Perceived discrepancies are caused by fundamental heterogeneity of study structure, questions, and technique, which make direct comparison of their findings challenging. The most prominent disparity relates to end points under investigation. The paper of Ma et al., for instance, considered vitamin A risk for poor pregnancy outcomes among an existing population of women with GDM, whereas others have examined its role in the initial development of GDM itself. These are distinct clinical questions. There also are significant methodologic differences; one group of researchers measured the level of vitamin A at a solitary point during mid-gestation, whereas the Xue et al. study employed a longitudinal observational design at all three trimesters. This is a crucial point as the role of vitamin A at the level of physiology and the metabolic requirement of the participant might vary during pregnancy. The divergent results might thus not be mutually exclusive, but rather, an illustration of the complex role of vitamin A at varying stages of GDM pathophysiology. Future investigations will have to employ standardized, prospective designs with clearly delineated end points and identical assay procedures if they are to clarify the role of vitamin A in GDM.

#### 2.3.2 Vitamin D

##### 2.3.2.1 Vitamin D and pancreatic β-cell function

Current clinical studies have clearly shown that Vitamin D deficiency during pregnancy has been a significant risk factor for GDM. Pregnant women with the lowest risk of GDM have serum vitamin D levels of 40–90 nmol/L (11). This firm clinical correlation can be explained due to the critical requirement of vitamin D for maintaining the health and function of pancreatic β-cells intact.

Another essential mechanism entails β-cell defense and survival. Research carried out by Mette observes that vitamin D exerts a positive effect on cell survival genes, anti-apoptotic genes, and inflammatory inhibition β-cell genes (18). In precise terms, as Chen has established, the VDR or vitamin D receptor pathway represses β-cell apoptosis prompted by the FoxO1 gene (13). That β-cell safeguarding system explains the clinical fact that the administration of vitamin D lowers glucose levels in the blood among females already harboring GDM (12), probably due to sparing their current cells which are capable of producing insulin from apoptosis.

Direct control of the secretion of insulin is also an essential process. There is inadequate secretion of insulin in GDM, which is calcium-dependent (15, 16). Vitamin D has its action on this process directly. Through its receptor, it opens calcium channels on β-cells, which provides the immediate stimulus for the release of insulin. A deficiency of vitamin D may therefore have a direct impact on β-cell secretion of adequate insulin to overcome insulin resistance caused due to pregnancy and so impair signaling of insulin (17). Finally, vitamin D possesses an essential anti-inflammatory role. Wang has remarked that deficiency will trigger the pro-inflammatory NF-κB signaling cascade (14). This leads to an influx of inflammatory cells in the pancreas and direct damage, reduced β-cell volume, and impaired function. This inflammatory damage provides another mechanistic rationale as for why there is value in maintaining adequate levels of vitamin D for preventing GDM β-cell dysfunction.

##### 2.3.2.2 Vitamin D and vascular endothelial function, inflammatory response, and oxidative stress

GDM typically accompanies systemic pathological processes, for instance, vascular endothelial dysfunction, resulting in prolonged insulin resistance. Vitamin D controls the process greatly by working on vascular factors like VEGF, sFlt-1, and PlGF (19). Whereas the clinical presentation may turn out complicated—with some studies showing greatly increased sFlt-1 and reduced PlGF among GDM patients (20), and others not showing any considerable level of sFlt-1 (21)—this emphasizes that the degree of damage on the endothelium may vary among GDM populations. All the same, vitamin D’s regulatory function on these pathways secures its critical function of sustaining vascular health intact in pregnancy.

Besides the vascular compartment, GDM includes placental inflammation and oxidative stress as central inducers of insulin resistance. Vitamin D counteracts these issues at the cellular level directly. For instance, Li demonstrated that the active version of vitamin D, 1,25(OH)2D3, inhibits the pro-inflammatory mTOR signaling cascade of trophoblast cells with consequent reduced central inflammatory cytokine concentrations of TNF-α and IL-6 (22). At the same time, vitamin D inhibits oxidative stress through regulation of the expression of antioxidant proteins as well as control of intracellular calcium homeostasis (19). Interestingly, the placenta of GDM itself may have an intrinsic defect in regulating vitamin D. Aberrantly expressed CYP2R1 and CYP27B1 metabolic enzymes have indeed been detected as causing insufficient local control of active vitamin D levels in GDM placentas, which could lead to localized deficiency as well as inflammatory responses (23). So, preventing insulin resistance seems to require treating downstream processes. Toward this purpose, Li further detected that the protein CAMK4 potently accelerates GDM-related insulin signaling with autophagic control (24), and so identifies CAMK4 as one of the drug targets for achieving cellular health restoration under this deleterious metabolic environment.

##### 2.3.2.3 Vitamin D and immune regulation

GDM is increasingly being viewed as an immune dysregulation disorder where excess inflammation has the ability to tip insulin resistance. Vitamin D has a key protective role where it is able to influence the immune system.

One of its key mechanisms is through the aid of regulatory T cells (Tregs), “peacekeepers” of the immune system due to their property of suppressing inflammation. Patients with GDM and their sera both contain low serum 25(OH)D3 and low expression of these vital anti-inflammatory cells. Direct associations have been observed in studies where the level of serum 25(OH)D3 is shown to be positively correlated with the percentage of CD4+ Foxp3+ regulatory Tcells within the peripheral blood. Note that this mechanistic link has immediate clinical ramifications: the very same study found that when the level of vitamin D declined, strong markers of insulin resistance—you guessed it: FIns (fasting insulin), HbA1c (glycosylated hemoglobin), and HOMA-IR (homeostasis model assessment of insulin resistance) levels—all increased (25). Here is what it implies: low levels of vitamin D result in diminished function of Tregs, hence amplified inflammation and worsening insulin resistance.

Further, vitamin D maintains healthy Th1/Th2 cell balance. Normal pregnancy is shifted toward inflammatory (Th2-predisposed) status, but it can be compromised within GDM. It has been discovered that patients with GDM’s low serum 25(OH)D3 levels can be related to a decrease of the percentage of Th2 cell and lead to a pro-inflammatory Th1/Th2 imbalance and overall immune suppression (26). Fostering both Tregs and proper Th1/Th2 status, the immune modulation of vitamin D is one of its strong mechanisms of precluding and treating GDM and supporting the clinician advice of elevating its supplementation.

##### 2.3.2.4 Vitamin D and lipid metabolism

GDM is tightly related to adipokine dysregulation, which is a disruption of hormones secreted from adipose tissue that results in insulin resistance. Clinical studies have characterized the individualized disruption within pregnancy. For instance, Gennaro found reduced levels of favorable adipokines, such as adiponectin (APN) and the soluble leptin receptor (sOB-R), are inversely correlated with the incidence of GDM. Direct correlations exist between higher levels of unfavorable adipokines, such as FABP4, chemerin, IL-6, and leptin and the odds of developing GDM (28).

Vitamin D appears prominently in this tale of metabolism. Indeed, it has been discovered that patients suffering from GDM have significantly low levels of both the vitamin D and of the protective adipokine APN, but their levels of chemokines are raised (27). This clinical observation literally draws together a link between vitamin D insufficiency and the aberrant adipokine profile of GDM.

Mechanistic justification of this link derives from preclinical evidence. Pich et al. found in a rat trial that treatment with vitamin D3 increased uterine APN levels directly and additionally upregulated gene expression of its receptors, Adipor1 and Adipor2 (29). This suggests that vitamin D plays a fundamental regulatory role itself in inducing the expression of beneficial adipokines. Overall, the information suggests a unifying narrative: GDM is a deleterious disruption of adipokine homeostasis, vitamin D is related to this disruption, and vitamin D itself has mechanistic capacity itself to reverse this homeostasis by increasing levels of protective adipokines like APN.

##### 2.3.2.5 Methods of vitamin D supplementation

Because of the central role of vitamin D in the maintenance of pancreatic β-cell function and regulation of the immune system and lipid metabolism, supplementation has become a key clinical intervention in GDM. Widespread clinical trial results are likely the direct result of those underlying biological mechanisms.

In pregnant women already diagnosed with pre-existing GDM, supplementation with vitamin D has been shown to improve both maternal and neonatal outcomes. One key meta-analysis verified that supplementation improves blood glucose and reduces rates of cesarean section, maternal hospitalization, postpartum hemorrhage, and diverse neonatal morbidity (12). It is further effective at correcting the disorders of lipid metabolism by reducing LDL and triglycerides and reducing cholesterol levels while increasing HDL (30). These clinical advantages can be appreciated as downstream effects of vitamin D improving β-cell survival, reducing system-wide inflammatory status, and correcting adipokine profiles.

From a preventive perspective, evidence confirms that adequate pre-pregnancy vitamin D status is essential before the development of major insulin resistance. It was found by Ma et al. that when serum 25(OH)D3 exceeds 50 nmol/L, the prevalence of GDM is markedly reduced (31). This favors an early or mid-pregnant supplementation program aimed at the prevention of the first β-cell injury and immune imbalances that may give rise to GDM.

Nonetheless, the timing and circumstances of supplementation are essential. That is the point of the observation that postpartum supplementation, although effective at elevating serum levels of vitamin D in GDM-affected mothers, did not enhance major markers of metabolism such as insulin resistance or HbA1c (32). This implies a “critical therapeutic window” within pregnancy itself when such benefits of metabolism may no longer occur. Analogously, a nested case-control study that lacked any raised risk of GDM associated with deficiency during early pregnancy perhaps because of sources of confounding such as inaccurate reporting or exposure to sunlight (33) highlights the difficulty of determining the period of greatest impact at intervention and one possibility is that it may occur at mid-gestation when the natural peak of insulin resistance is seen.

Despite strong and discordant pathophysiologic evidence for GDM and vitamin D, clinical literature presents a less definitive and more complex portrayal. A more detailed examination of evidence offers insights into the timing of deficiency and supplementation as indicators of the outcome. For example, although robust evidence suggests that supplementation benefits glucose control during pregnancy, there is no long-term glucose marker advantage associated with postpartum supplementation. This implies there is an inherent therapeutic range for the metabolic effects of vitamin D that is diminished at birth. Similarly, the possibility that prenatal deficiency at or around the diagnosis of GDM may not increase risk does not negate its importance; rather, it suggests a more complex hypothesis: the critical period for vitamin D’s influence likely occurred during mid- and late-gestation, when natural increases in the risk of insulin resistance are at their peak. The ambiguous findings regarding biomarkers like sFlt-1 indicate that pathological processes, such as endothelial function, differ among patient groups with GDM. Consequently, the primary inquiry has shifted from whether vitamin D has any function to asking how, when, and for whom it is relevant. Future studies must progress past merely identifying associations and should focus on determining optimal timing for administration, dosing, and patient demographics for conclusive intervention.

#### 2.3.3 Vitamin E

Vitamin E is a potent antioxidant. A large-scale retrospective cohort study conducted by Shi (34) found that women with lower levels of vitamin E during early pregnancy had a higher risk of developing GDM. Specifically, women with early pregnancy vitamin E concentrations below 7 mg/L exhibited an “L-shaped” relationship between early pregnancy vitamin E levels and GDM risk, with the inflection point around 7 mg/L, which is significantly higher than the clinical threshold for vitamin E deficiency, defined as 5.0 mg/L. The study suggested that maintaining vitamin E levels above 7 mg/L during early pregnancy may help reduce the risk of GDM.

Additionally, Ma (9) identified vitamin E as an independent risk factor for adverse pregnancy outcomes in GDM patients (P<0.05). They proposed that elevated serum vitamin E levels in GDM patients might contribute to impaired pancreatic β-cell function. Zhou (35) found that excessively high vitamin E levels during mid-pregnancy were a risk factor for GDM (aOR=1.640, 95% CI: 1.316-2.044). Moreover, maternal serum vitamin E concentrations during early and mid-pregnancy were positively correlated with the occurrence of GDM (early pregnancy: aOR=1.056, 95% CI: 1.038-1.073; mid-pregnancy: aOR=1.062, 95% CI: 1.043-1.082).

The evidence presented on vitamin E reveals a complex, non-linear relationship with GDM that defies a simple interpretation of deficiency or excess. A critical synthesis of these seemingly contradictory findings suggests a U-shaped or J-shaped risk curve, where both low and high concentrations of vitamin E are associated with adverse outcomes. The study by Shi et al. establishes the risk associated with deficiency in early pregnancy, while the findings from Zhou et al. highlight the danger of excess in both early and mid-pregnancy. This paradox may be rooted in vitamin E’s dual role as an antioxidant at physiological levels and a potential pro-oxidant at supra-physiological concentrations, particularly within the high-stress metabolic environment of GDM. Furthermore, the studies investigate different clinical endpoints: Shi and Zhou focus on the incidence of GDM, whereas Ma examines adverse outcomes in patients already diagnosed with the condition. These findings are not necessarily mutually exclusive; rather, they suggest that maintaining vitamin E within a narrow therapeutic window is crucial throughout gestation. An elevated level could be both a risk factor for developing GDM and a contributor to poor outcomes once the disease is established. Future research must therefore move beyond simple correlation and aim to define this optimal physiological range, questioning whether elevated vitamin E is a causal factor or merely a biomarker for other dietary or metabolic disturbances.

## 3 Water-soluble vitamins and GDM

### 3.1 One-carbon metabolism vitamins

One-carbon metabolism involves vitamins such as vitamin B6, folate (vitamin B9), and vitamin B12, and abnormalities in this metabolic pathway may be linked to the development of GDM. A prospective cohort study by Saravanan (36) found that lower vitamin B12 levels during early pregnancy were associated with higher fasting blood glucose levels. This was also linked to increased blood glucose levels at 2 hours after an OGTT. Moreover, lower vitamin B12 levels were shown to increase the risk of GDM. Folate levels were positively correlated with GDM risk, meaning that higher folate levels were associated with elevated 2-hour post-glucose levels and an increased risk of GDM. However, the study had certain limitations, including a small sample size and wide confidence intervals in its ethnic subgroup analysis.

Betaine, a key methyl donor in the one-carbon metabolic pathway, participates in the remethylation of homocysteine to form methionine. Gong (37) found that higher plasma betaine levels in twin pregnancies were significantly associated with a lower risk of GDM. They further suggested that folate, as another crucial nutrient in one-carbon metabolism, influences homocysteine levels and, thus, GDM risk. The interaction between betaine and folate in the one-carbon pathway highlights potential nutritional interventions that could play an important role in preventing GDM.

Vitamin B3, in the form of nicotinamide (Nam), is a precursor to nicotinamide adenine dinucleotide (NAD+), an essential coenzyme in one-carbon metabolism. A prospective cohort study by Tranidou (38) found that excessive intake of niacin during pregnancy increased the risk of GDM. The study proposed that niacin, as a coenzyme in redox reactions within the one-carbon metabolic pathway, could influence insulin secretion and, consequently, glucose metabolism.

The findings related to the vitamins engaged in one-carbon metabolism offer a fascinating and complex narrative that disputes conventional views, particularly about folate. A detailed assessment shows that the comprehensive integration of the metabolic pathway, instead of the individual levels of any particular vitamin, is the most effective way to sustain glucose homeostasis during pregnancy. The notable inverse connection between elevated folate levels and a heightened risk of GDM is particularly important and deserves consideration. This may not directly condemn folate, but it highlights the risks associated with high levels of synthetic folic acid found in supplements, which can overload metabolic functions and lead to the accumulation of unmetabolized folic acid (UMFA), possibly masking current vitamin B12 deficiencies—a known risk factor for GDM. In this situation, the positive effects of vitamin B12 and betaine, along with the negative effects of too much niacin, reinforce the “metabolic balance” theory. B12 and betaine support the crucial remethylation of homocysteine, while excessive niacin can disrupt redox balance, highlighting the pathway’s susceptibility to both deficiency and excess. Although the limitations of the referenced studies, such as sample size, require careful consideration, these results collectively shift focus from the supplementation of particular nutrients to the essential need for keeping related one-carbon nutrients in balanced harmony with one another.

### 3.2 Vitamin C

Oxidative stress is one of the mechanisms underlying the pathogenesis of GDM. As an antioxidant, vitamin C may play a role in the prevention of GDM. Current research has confirmed that insufficient vitamin C intake during pregnancy significantly increases the risk of GDM (39). Liu (5) analyzed that higher dietary vitamin C intake during mid-pregnancy was associated with a lower risk of GDM, with a daily intake of more than 200 mg of vitamin C potentially offering a protective effect against GDM. However, studies examining the relationship between total vitamin C intake and GDM risk in patients remain controversial. Liu (5) did not find a significant correlation between the two. Furthermore, Zhou (39) pointed out that the relationship between vitamin C and GDM might differ under different diagnostic criteria for GDM. According to the IADPSG (International Association of Diabetes and Pregnancy Study Groups) criteria, GDM can be diagnosed if any one of the results from the OGTT exceeds the threshold, whereas the ADA (American Diabetes Association) criteria require two or more elevated readings. Therefore, under the ADA criteria, women diagnosed with GDM experience suffer more severe oxidative stress, and their vitamin C levels are more significantly reduced.

In addition, due to limited dietary intake in GDM patients, particularly in late pregnancy, there is a risk of iron deficiency anemia, which can lead to adverse pregnancy outcomes such as preterm birth (40). Preventing iron deficiency anemia during the management of blood glucose in GDM pregnancies is crucial. Some clinical studies on iron deficiency anemia have shown that vitamin C can enhance the absorption of iron by reducing ferric iron (Fe3+) to ferrous iron (Fe2+), thereby promoting the formation of soluble iron compounds. This process alleviates the symptoms of anemia. A study by domestic scholars found that pregnant women with mild to moderate iron deficiency anemia who received oral iron sulfate (200 mg, tid) combined with vitamin C (100 mg, qd) had a higher total effective rate than those who received only oral iron sulfate (300 mg, tid), and the incidence of adverse pregnancy outcomes was lower in the experimental group than in the control group (P<0.05) (41). This indicates that the combination of vitamin C with iron supplementation can effectively enhance iron absorption. However, this study only lasted for 2 months and lacked follow-up data on long-term efficacy and safety, which makes it difficult to assess the long-term effects and potential adverse reactions. Additionally, the study was a single-center clinical trial involving only 298 patients, which could affect the statistical significance of the results. Future studies should increase sample size, conduct multi-center trials, and design long-term follow-ups to improve the scientific rigor and credibility of the results. Further research on the correlation between vitamin C and iron-deficiency anemia in GDM patients, as well as determining the optimal timing and dosage of vitamin C supplementation, is warranted.

The clinical data regarding vitamin C and GDM, though encouraging, is marked by considerable complexity that requires thorough assessment. The link between vitamin C and GDM risk differs depending on the diagnostic test applied, underscoring an important aspect: it suggests that vitamin C’s antioxidant function may not universally offer protection but instead becomes relevant in situations of greater metabolic disturbance and increased oxidative stress, as specified by the more rigorous ADA standards. Moreover, the gap between protective vitamin C dietary consumption and conflicting findings regarding total intake challenges the idea of universal advantages and instead emphasizes the complex relationships among nutrients, where benefits may be tied specifically to the food matrix or impacted by additional factors linked to supplementation. Although its effectiveness in preventing GDM is contested, vitamin C’s significant role in boosting iron absorption positions it as an excellent therapeutic addition for addressing complications related to GDM, such as anemia. In carrying out this dual role, the clinical importance of vitamin C might be most evident not in stopping GDM, but in mitigating the harmful effects for patients within the GDM range.

## 4 Limitations

This analysis highlights growing evidence linking vitamin levels to GDM, while also pointing to major shortcomings in current research. Many studies rely on observational designs, making it difficult to prove cause-and-effect and to rule out confounding factors such as diet, lifestyle, and socioeconomic status. Differences in methods, small sample sizes, and single-site trials further weaken conclusions, as seen in the vitamin C and one-carbon metabolism studies.

Another major issue is inconsistent GDM diagnostic criteria. Results differ when using IADPSG standards compared to ADA guidelines, limiting comparability and complicating meta-analyses. Geographic, ethnic, and dietary variations also add complexity, as nutrient baselines differ widely across populations.

Together, these flaws underscore the need for large, multicenter randomized controlled trials. Future studies must use uniform diagnostic criteria and systematically account for confounders. Only with this rigor can we clarify how vitamins influence the onset and outcomes of GDM.

## 5 Discussion

This review discusses how vitamin status influences risk of GDM. Deficiency and excess of certain vitamins can adversely affect glucose regulation in pregnancy, although findings often vary due to differences in study design and participants. Rather than reviewing each vitamin in sequence, it is valuable to think of them as part of a bigger picture that ties diet, metabolism, immunity, and oxidative stress together. A summary of the overall associations, evidence strength, and key limitations across vitamins is presented in **Table 1**.

**Table 1:** Vitamins and metabolic pathways in gestational diabetes mellitus: evidence and limitations.

Vitamin/Pathway	Overall association with GDM	Evidence strength (this manuscript set)	Key limitations
Vitamin D (11, 12, 31–33)	Deficiency consistently associated with ↑ GDM risk; supplementation improves glycemic control and selected maternal–neonatal outcomes	High (multiple meta-analyses, RCTs, prospective cohorts)	Heterogeneity in diagnostic criteria and assay cut-offs; variability in supplementation timing and dosage; limited long-term follow-up data
Vitamin E (9, 34, 35)	Non-linear (U- or J-shaped) association: both low and high levels linked to increased GDM risk or adverse pregnancy outcomes	Moderate (large retrospective cohorts, observational studies)	Inconsistent study endpoints (incidence vs outcomes in GDM patients); residual dietary/metabolic confounding
Vitamin A (6, 9, 10)	Conflicting findings: some studies suggest protective effect, others show no significant association	Low–moderate (retrospective and longitudinal observational studies)	Heterogeneity in design, timing of vitamin measurement (trimester-specific), and outcome definitions
Vitamin B12/Folate (B9) (36)	Low B12 associated with ↑ GDM risk; high folate associated with ↑ risk, possibly due to unmetabolized folic acid and interaction with B12 deficiency	Moderate (prospective cohort data)	Dietary and ethnic confounding; imbalance in folate fortification/supplementation; limited mechanistic clarity
Betaine (37)	Higher plasma betaine associated with ↓ GDM risk, likely through homocysteine remethylation	Low–moderate (observational data, twin pregnancies)	Limited generalizability beyond twin gestations; relatively small sample sizes
Niacin (B3) (38)	Excessive intake associated with ↑ GDM risk, potentially through altered redox balance and impaired insulin secretion	Low–moderate (prospective cohort)	Dietary intake misclassification; threshold for “excess” remains unclear
Vitamin C (5, 39, 41)	Higher dietary intake may reduce GDM risk; total intake findings inconsistent; may enhance iron absorption and mitigate anemia in GDM	Low–moderate (cohort studies, small RCTs)	Differences in diagnostic criteria (IADPSG vs ADA); short study durations; limited sample sizes

Vitamin D is also the single best predictor of GDM. Reduced status injures beta-cell function of the pancreas, augments inflammation, and injures vessels. Supplement studies show beneficial vitamin D influences on glucose in blood and maternal-fetal variables, in spite of issues of timing. Evidence favors mid-pregnancy as maybe the best time, superior to initiation in late pregnancy.

Vitamin E has a more complex picture. Research displays a U- or a J-profile: too little or too much increase risk. Within normal range, vitamin E has antioxidant action, providing protection against stress and cellular harm. At very high doses, though, it might convert to a pro-oxidant behavior, worsening metabolic stress. This ambivalence is likely to explain discordant results in research. The take-home: it’s about balance, and more is not necessarily better. The role of vitamin A in gestational diabetes is controversial. Protective functions against oxidative stress and β-cell failure have been shown in some studies, while in some, there is no significant association. The controversial results may occur because of what stage of gestation vitamin A is measured. Due to alterations in metabolic requirements across trimesters, its role may also vary across gestational age. This necessitates setting up standard longitudinal studies to define its role.

One-carbon metabolism water-soluble B vitamins—folate, B12, and B6—demonstrate a fragile interconnection. Low B12 is linked with augmented GDM risk, while higher intakes of folate, in a seeming reversal, are associated with impaired glucose tolerance. The argument is that unmetabolized folic acid might mask B12 deficiency. Hence, relative B/B12 equipoise, more so than absolute intakes per se, seemed critical in establishing metabolic stability in gestation. Betaine provides added protection, further underscoring regulated coordination of this pathway. In contrast, higher intakes of niacin may interfere with redox equilibrium and insulin secretion, raising GDM risk. The consequences of these findings are that interactions of nutrients, more so than solitary vitamin action, seem critical.

Vitamin C has been heavily studied because of its antioxidant properties as well as its ability to increase iron absorption. Some studies show higher intake lowers GDM risk, although this conclusion changes when looking at total intake. The variability due to diagnostic thresholds, such as use of IADPSG compared to ADA guidelines, further confounds this. However, vitamin C supplementation has been successful in raising iron absorption as well as reducing anemia, a common issue in GDM. Despite its immediate effect upon GDM being in question, it might help in improving maternal outcome in general.

Overall, data show a complicated picture: vitamin effects are not invariant, nor are they equivalent. Endpoints differ across nutrient, time of pregnancy, and mother’s background. Heterogeneity in research design—diagnostic thresholds, sample sizes, and uncontrolled confounders such as diet, ethnic background, and socioeconomic status—further confounds interpretation.

Future research must be large, multicenter randomized controlled studies that are mindful of confounders and utilize standardized diagnostic protocols. Mechanistic studies should also be performed to establish causal mechanisms, including oxidative stress, immune response, activity of adipokines, and one-carbon metabolism. Precision medicine strategies, accounting for maternal genetics, baseline lifestyle, and nutrition, might also hold value in identifying women that will benefit most from individually focused vitamin supplementation.
